# Cost-Minimization Model of a Multidisciplinary Antibiotic Stewardship Team Based on a Successful Implementation on a Urology Ward of an Academic Hospital

**DOI:** 10.1371/journal.pone.0126106

**Published:** 2015-05-08

**Authors:** Jan-Willem H. Dik, Ron Hendrix, Alex W. Friedrich, Jos Luttjeboer, Prashant Nannan Panday, Kasper R. Wilting, Jerome R. Lo-Ten-Foe, Maarten J. Postma, Bhanu Sinha

**Affiliations:** 1 Department of Medical Microbiology, University of Groningen, University Medical Center Groningen, Groningen, the Netherlands; 2 Certe Laboratory for Infectious Diseases, Groningen, the Netherlands; 3 Department of Pharmacy, Unit of PharmacoEpidemiology & PharmacoEconomics, University of Groningen, Groningen, the Netherlands; 4 Department of Clinical Pharmacy and Pharmacology, University Medical Center Groningen, Groningen, the Netherlands; 5 Department of Epidemiology, University Medical Center Groningen, Groningen, the Netherlands; Johns Hopkins Bloomberg School of Public Health, UNITED STATES

## Abstract

**Background:**

In order to stimulate appropriate antimicrobial use and thereby lower the chances of resistance development, an Antibiotic Stewardship Team (A-Team) has been implemented at the University Medical Center Groningen, the Netherlands. Focus of the A-Team was a pro-active day 2 case-audit, which was financially evaluated here to calculate the return on investment from a hospital perspective.

**Methods:**

Effects were evaluated by comparing audited patients with a historic cohort with the same diagnosis-related groups. Based upon this evaluation a cost-minimization model was created that can be used to predict the financial effects of a day 2 case-audit. Sensitivity analyses were performed to deal with uncertainties. Finally, the model was used to financially evaluate the A-Team.

**Results:**

One whole year including 114 patients was evaluated. Implementation costs were calculated to be €17,732, which represent total costs spent to implement this A-Team. For this specific patient group admitted to a urology ward and consulted on day 2 by the A-Team, the model estimated total savings of €60,306 after one year for this single department, leading to a return on investment of 5.9.

**Conclusions:**

The implemented multi-disciplinary A-Team performing a day 2 case-audit in the hospital had a positive return on investment caused by a reduced length of stay due to a more appropriate antibiotic therapy. Based on the extensive data analysis, a model of this intervention could be constructed. This model could be used by other institutions, using their own data to estimate the effects of a day 2 case-audit in their hospital.

## Introduction

Inappropriate and inefficient use of antimicrobial therapy and consequent resistance development can lead to unwanted clinical effects, such as toxicity, hospital acquired infections, longer length of stay [1] and to high costs for hospitals and society [2–4]. In 2007, the extra in-hospital costs for antibiotic resistance were estimated at 900 million Euros for the European Union, and another 1.5 billion Euros societal cost [5], although estimations on societal costs are thought to be underestimated [6]. Without appropriate action, resistance rates will rise, as will the subsequent costs [7]. These unwanted financial consequences are becoming more and more relevant in times of healthcare cost reductions and savings programs [8]. To control these costs, it is critical to cut spending wisely by focusing on inefficient services [9] such as inadequate antimicrobial therapy. Improving this treatment can not only have a positive effect on patient care and safety, but also be a potential contributor to wise budgetary savings.

One way to improve patients’ antimicrobial therapy and stimulate prudent use is through Antimicrobial Stewardship Programs (ASPs) [10–12]. This term covers a wide arrange of interventions, all done with the objective to optimize patients’ antimicrobial therapy. Several papers discussed the financial effects of ASPs, and although difficult to compare due to the wide arrange of different interventions in different populations and because the quality of such evaluations is sub optimal, the consensus seems to be that costs can be saved [13–16].

Part of an ASP is an Antibiotic Stewardship-Team (A-Team). At the University Medical Center Groningen (UMCG) in the Netherlands, an A-Team has been implemented at a urology ward to perform a case audit after 48 hours (day 2) of initiation of antibiotic therapy to improve the quality of care. The aim of this study was to construct a cost-minimization model reflecting direct costs and benefits on a hospital-wide level. This has been achieved by means of a historic cohort study for this case-audit performed by an A-Team, with the focus on direct return of investment from a hospital perspective. This study provides a framework, which can be used for other hospitals when implementing an A-Team, giving an indication what the direct return on investment of this specific ASP would be.

## Material and Methods

### Antibiotic Stewardship Team

The day-2 case audits by the A-Team were implemented within a 1339-bed academic medical center. It is a multidisciplinary team consisting of clinical microbiologists, infectious disease specialists and hospital pharmacists. One of the team members performed ward visits and discussed patients’ antibiotic therapy with the attending physician (fellow/resident). These ward visits could include a bed-side consultation and examination depending on the patient’s condition. During weekends, the consultations were either done by phone or on site during the subsequent working day. Eligible patients were selected on the basis of an automatic e-mail alert from the hospital’s pharmacy. An alert, generated from a clinical rule program (Gaston Medecs BV, Eindhoven, the Netherlands) was sent when a patient used an antibiotic, considered to be of specific relevance (flucloxacillin, amoxicillin/clavulanic acid, piperacillin/tazobactam, cefuroxime, ceftriaxone, meropenem, clindamcyin, tobramycin, ciprofloxacin, vancomycin and teicoplanin), beyond 48 hours after initiation. Together with the attending physician, interventions were decided upon to improve the therapy. The case-audits were implemented on a urology ward after a successful test on a trauma surgery ward [17].

### Patients and historic control cohort

Patients who stayed at the urology ward and received consultations by an A-Team member, between June 2013 and June 2014 were evaluated. Patients of whom antibiotics were started within three days of admission were included in this financial evaluation, resulting in data for 114 included patients. Financial effects related to length of stay, antibiotic consumption and nursing time needed for intravenous treatment were compared to a frequency-based historic cohort. The control cohort included patients who stayed at the same ward within a period of 30 months before the intervention started. This cohort was filtered based on the frequency of the Dutch equivalent of Diagnosis Related Group (DRG) codes of the intervention patients (DBC, http://www.dbconderhoud.nl) and the same antibiotics for 48 hours consecutively. Treatment policies based on national and local guidelines did not change within this period.

To account for the modifying effect that patients’ indications can have, subgroups were analyzed on the basis of their DRG codes. The first group (DRG Group 1) consisted of patients with codes for infections and infections-related indications. The second group (DRG Group 2) consisted of patients with codes for more severe underlying diseases (e.g. cancer), but who developed a subsequent clinical infection and received antibiotics.

### Economic data

All prices are given in Euros, 2013 level. Older prices were adjusted to 2013 using the Dutch consumer price index (statline.cbs.nl). Costs for the implementation and running of the A-Team were subdivided into personnel costs of the A-Team, costs made at the ward, medical costs, research/evaluation costs, overhead and implementation costs. Personnel costs are based upon Dutch gross salaries. Costs per hour for medical specialists in the A-Team, the hospital pharmacist, attending physicians, nurses and investigators were based on gross salaries and set at 120, 120, 35, 30 and 25 Euros respectively [18]. The A-Team member scored total time for every consultation, including administration, this time has been used for the cost calculations. Because no new personnel were hired for this project, fixed costs did not change. Following the principle of opportunity costing we therefore used these measured times as indication for the opportunity costs that occurred for the A-Team member and the attending physician.

Prices for the antibiotic medication were provided by the hospital pharmacy and reflect the actual prices charged by pharmaceutical companies. For all data, 2013 prices were used to account for possible changes over time.

Costs of one patient day in the hospital were based on the internal costing system applied to the urology ward, based on the actual budgets of 2012, resulting in a total of €716 per patient per day. Notably, overhead costs (including building costs, maintenance, equipment, personnel costs for daily care, etc) are included in these estimations; procedures are not included in this figure because we believe that a reduction in LOS does not influence the number of procedures substantially.

### Analyses

Subsequently, total costs and benefits of this specific A-Team case-audit on day 2 were compared via a model. Costs for the consultations were subtracted from the costs of the chosen outcome measures, if they statistically significantly differed from the historic control cohort. For the model, values were calculated using the historic cohort. The respective variables are shown in brackets.

It was assumed that outcome measures of patients having a DRG for an infection related problem could be influenced, but that patients with (severe) underlying causes will not see any differences in the outcome measures. This ratio was therefore included in the model as the percentage of patients with infection-related DRG codes (*P*_*DRG*_).

A change in hospital days (Δ*LOS*) was calculated and multiplied by the abovementioned price (*C*_*bed*_).

A switch form IV to oral administration is one of the interventions that was promoted by the A-Team. Therefore, nursing time spent on the administration of IV treatment per patient (*T*_*nurse*_) was calculated for this study, based on a survey on the ward. *P*_*IV*_ denotes the percentages of patients that received IV treatment. This number was multiplied by the nursing time and the gross salary of the nurse.

Case-audit costs (*C*_*audit*_) were calculated based on time spent by the A-Team doctor for the case-audit. For each consultation he/she scored the total time spent, including travel time and administration. Costs were multiplied by the average number of audits per patient (*n*_*audit*_) and the gross salary. The total figure also included costs for the attending physician on the ward, the hospital pharmacist, an investigator of the department and miscellaneous costs (e.g. meetings of the A-Team members).

Lastly, the average costs of the antimicrobials per patient (*C*_*ab*_) were evaluated between the group that received consultations and the historic cohort group.

This resulted ultimately in the following formula for the costs and benefits of the A-Team:

$$Revenue\;=\;P_{DRG}\left(\left(\Delta LOS\times C_{bed}\right)+\left(P_{IV}\times T_{nurse}\times C_{nurse}\right)+\;C_{ab}\right)-\;\left(C_{audit}\times n_{audit}\right)$$

For the calculation of a return on investment (ROI), the total number is divided by the total costs, giving a ratio for the direct return that can be expected for the hospital. Finally, for the parameters with the most impact on the model, deterministic sensitivity analyses were performed. Furthermore, for a set of parameters, a probabilistic sensitivity analysis (PSA) was performed. For this purpose, 2500 repeat drawings were done.

### Ethics statement

The described stewardship case-audit was normal every day care implemented within the hospital and approved by the antimicrobials committee following national guidelines. ASPs are mandatory by Dutch law for every hospital. Data was collected retrospectively from the hospital's data warehouse. It was anonymous and did not contain any (in)directly identifiable personal details. Following Dutch legislation and guidelines of the local ethics commission, approval was therefore not required (http://www.ccmo.nl).

### Statistic analysis

Depending on the type of data, appropriate tests were applied. Due to the analyses on two subset groups, the significance threshold was set at p < 0.025 to account for possible familywise error rates. Analysis was done with IBM SPSS Statistics 20 (IBM, Armonk NY, USA).

## Results

Firstly the implementation costs were calculated. To then examine the costs or benefits of the ASP, all parameters for the model that were unknown were determined, based on the comparison between the patients with interventions and the patients without intervention in the historic cohort. To account for uncertainties that the model might possess, multivariate sensitivity analyses and a probabilistic sensitivity analysis were performed. Ultimately the final number was calculated with the model.

### Implementation costs

Before starting the A-Team there was time invested to develop the procedures and protocols that were used, as well as the developing and testing of the clinical rule of the e-mail alerts. Time was scored retrospectively based upon A-Team members’ personal time schedules. Total costs came to €17,732. This includes implementation for other wards as well. These costs are not used in the model, because they are independent of this specific ward.

### Model parameters

All parameters in the model were calculated using the compared data of the patients with and without interventions. Total compliance of the interventions was 92.1%. For the two DRG groups, only in DRG Group 1 significant effects were found. For this ward, 61% of the patients were part of this group (*P*_*DRG*_). All measured effects were thus multiplied with this percentage. Within DRG Group 1, LOS (Δ*LOS*) dropped from 7.57 (95% CI: ±0.65) to 6.20 (95% CI: ±0.61) (p = 0.012). Nursing time needed for the administration of IV antibiotics (*T*_*nurse*_) was based upon a survey at the specific urology ward. IV-treatment was assessed to cost on average 64.83 minutes a day. Respective times were 10.5 minutes for preparation per dosage; one time 7.5 minutes for catheter insertion; 10 minutes every 4 days for changing the catheter; 10 minutes for control per day; one time 2.5 minutes for removal. On average, IV antibiotics were given 2.27 times a day for 4.04 days. For DRG Group 1 the nursing time dropped from 251.66 minutes (95% CI: ± 32.94) to 178.19 (95% CI: ±40.91) (p = 0.016). 74% of the audited patients received IV treatment (*P*_*IV*_). Within DRG Group 2 there were no significant changes (Table 1).

**Table 1:** Model parameters.

		Intervened	Control cohort	P-value
DRG Group 1	Total patients	70 (61.40%)	209 (58.54%)	
	Age	55.25 (± 3.71)	59.35 (± 2.25)	0.046^a^ ^,^ ^e^
	Male	51%	60%	0.112^b^
	Mean LOS	6.20 (± 0.61)	7.57 (± 0.64)	**0.012** ^c^
	IV patients	52 (74.29%)	168 (80.38%)	
	Mean IV nursing time	178.19 (± 40.91)	251.66 (± 32.94)	**0.016** ^d^
	Antibiotic costs	€ 13.34 (± € 4.54)	€ 17.42 (± € 3.33)	0.030^a^
	Mean number of consultations	1.11	-	
DRG Group 2	Total patients	44 (38.60%)	148 (41.46%)	
	Age	61.12 (± 4.49)	64.57 (± 2.62)	0.139^a^
	Male	75%	84%	0.708^b^
	Mean LOS	8.36 (± 1.26)	8.10 (± 0.87)	0.801^c^
	IV patients	32 (72.73%)	114 (77.07%)	
	Mean IV nursing time	206.70 (± 67.72)	249.66 (± 40.45)	0.550^d^
	Antibiotic costs	€ 15.76 (± € 8.30)	€ 21.47 (± € 6.56)	0.637^a^
	Mean number of consultations	1.09	-	

Mean effects per patient, analyzed per subgroup. Age in years, length of stay (LOS) in days, IV nursing time in minutes and when applicable 95% CI or percentage is shown in brackets. P-values < 0.025 were considered significant to account for familywise error rates.

^a)^ Mann-Whitney U test

^b)^ Chi square test

^c)^ Log-Rank test (Mantel Cox)

^d)^ Unpaired, two-sided t-test

^e)^ No correlating influence of age was found

Cost for 1 case-audit was on average €80.26 (*C*_*audit*_). 1 audit took on average 12.1 minutes (95% CI: ± 0.77 min.), costing €24.03 (95% CI: ± €1.53). During the audit, the A-Team member spoke with the attending physician, costing €7.01 (95% CI: ± €0.45). The A-Team’s hospital pharmacist was responsible for the e-mail alerts and the clinical rules, including daily maintenance. His costs were estimated at €35.95 per audit. An investigator of the department evaluated the A-Team interventions for quality assurance, costing €2.08 per audit. This amounts to a total of €69.07 of direct personnel costs of the A-Team. Furthermore there are miscellaneous costs that need to be taken into account (e.g. meetings of A-Team members); total costs of this are €11.19 per audit. On average, patients received 1.09 case-audits (*n*_*audit*_) per admission.

Mean costs per patient for antimicrobial medication (*C*_*ab*_) showed a trend towards decreasing costs compared to the historic control groups, however it was not significant. In DRG Group 1 mean costs decreased from €17.42 (95% CI: ± 3.33) to €13.34 (95% CI: ± 4.55) (p = 0.030), for DRG Group 2 costs went from €21.47 (95% CI: ± 6.56) to €15.76 (95% CI: ± 8.30) (p = 0.637). However, because p-values were higher than 0.025 effects on cost price were not taken into account in the model (Table 1).

### Patients’ main diagnosis and price of a hospital day had the largest impact on the total benefits

**Fig 1:**
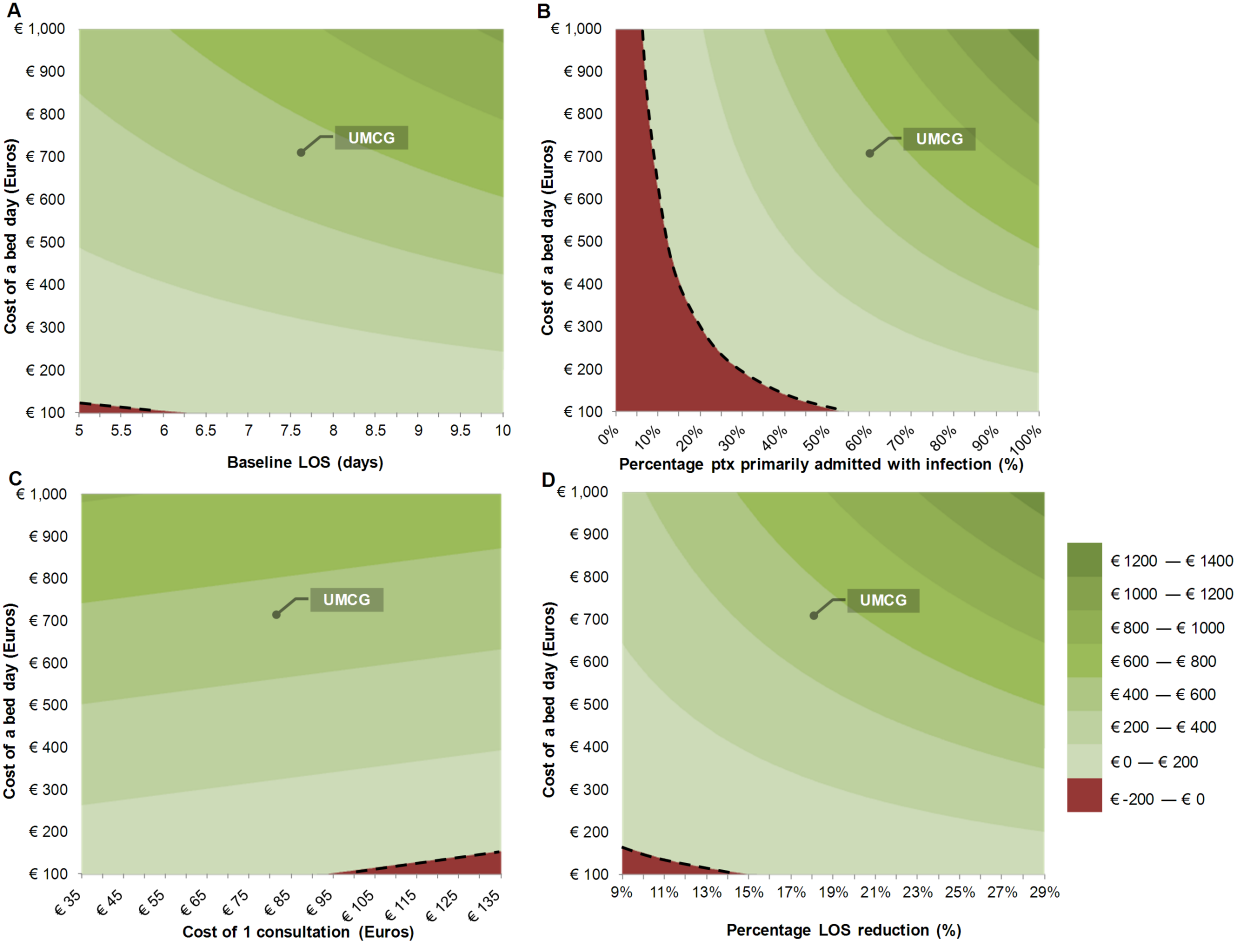
Surface graphs showing different parameter ranges. All graphs show a range of the hospital day price on the Y-axis with another parameter on the X-axis. The color represents the costs or benefits ranging from minus €200 in dark red to €1400 in dark green; the dashed line indicates €0. The total value for the UMCG, using the urology study values is shown in every plot. A: Range of baseline average of LOS before starting with an A-Team. B: Range of percentage of patients primarily administered with an infection. C: Range of costs of 1 consultation. D: Range of percentage of expected LOS reduction through A-Team interventions.

For the parameters with the largest/highest effect on the end result, multivariate sensitivity analyses were performed to visualize the effect they have on the return of investment. On the surfaces plots (Fig 1) the ranges in Euros are shown. Besides the price for a hospital day, the distribution of patients with or without severe underlying problems (*P*_*DRG*_) had the highest impact on the financial profitability.

### Probabilistic Sensitivity Analysis (PSA)

**Fig 2:**
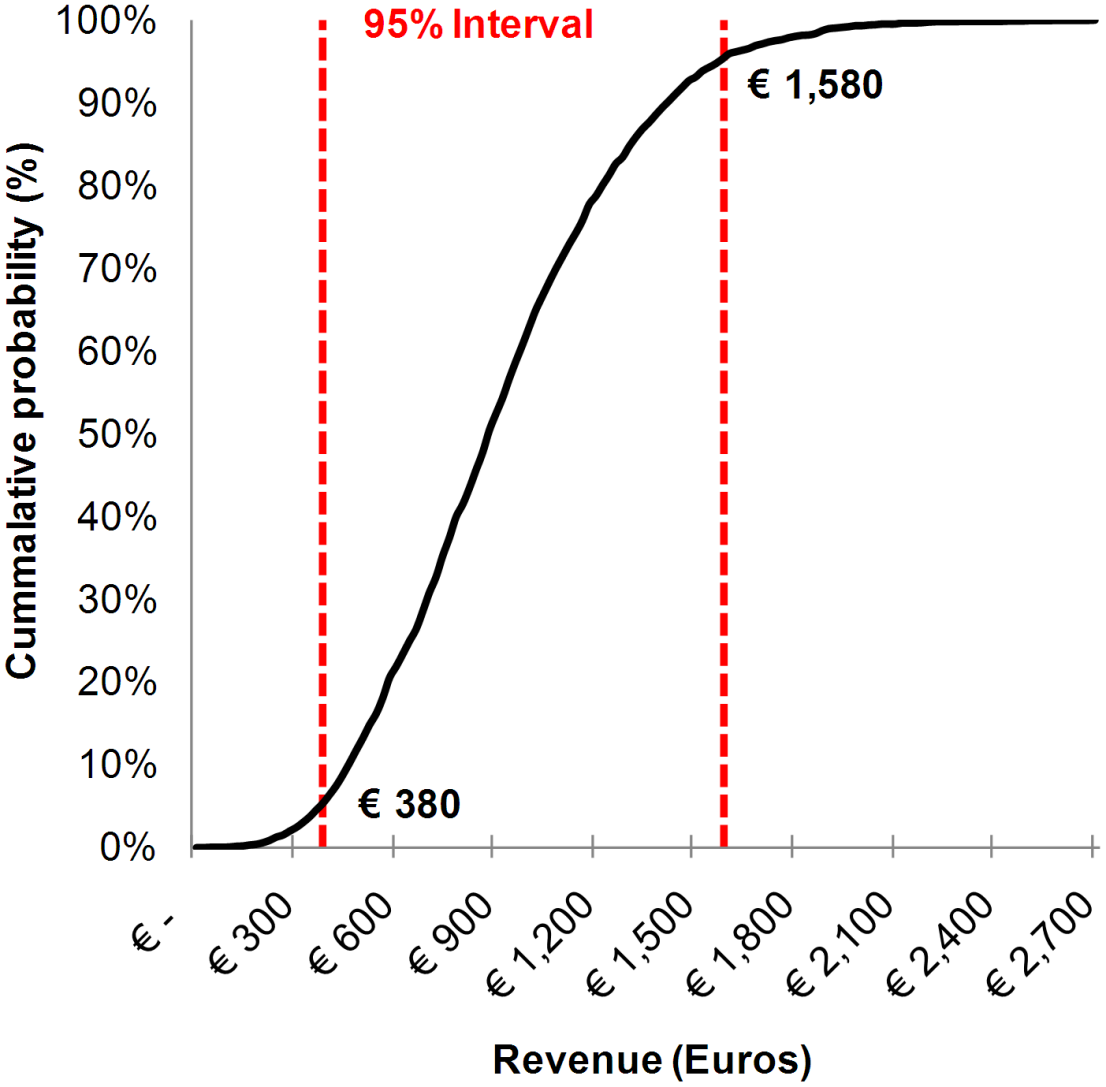
Probabilistic Sensitivity Analysis for DRG Group 1. Depicted is the probability of the costs or benefits in Euros with a PSA done for LOS, nursing time and antibiotic costs. The model ran for 2500 repeats. The 95% confidence interval is represented with the two dashed lines.

In the model for DRG Group 1, where significant results were found, a PSA was performed for LOS, nursing time and antibiotic costs. Other parameters within the model had the baseline value (Table 1). For percentage of patients primarily administered with an infection baseline value was set at 100%. The PSA gave an expected total benefit between €32 and €2,696, with a median of €877 and a 95% interval between €380 and €1,580 per patient (Fig 2).

### The hospital saved considerable amounts of money with their A-Team

Costs and benefits for the hospital based on the implementation on the urology ward were calculated with the model. Using all the baseline values as mentioned above, for one year of interventions and 114 patients the total profits amounted to €60,306 (€529.05 per consultation), which led to a direct return on investment (ROI) of 5.9.

## Discussion

Here we show a model, which can be used to calculate the financial effects of an A-Team. Using the data from the intervention study, the case-audit on day 2 performed by an A-Team was cost-effective and saved more than 60,000 Euros with consulting just 114 patients in one year on one ward. Revenues can therefore be expected to be much higher when a program like the one presented here were implemented in the whole hospital while implementation costs would remain relatively stable and acceptable.

Antimicrobial stewardship programs (ASP) are introduced in more and more hospitals to improve quality of antimicrobial therapy and thereby controlling the growing resistance to antibiotics. Consequently more studies are published about the effects of stewardship programs, both with regard to clinical and financial aspects [10]. In our hospital, an ASP was already in place and it has been expanded with an A-Team doing case-audits on day 2.

This study looked at two outcome measures (LOS and antibiotic use). The effects on those parameters will be greater for patients who were admitted primarily for an infection (DRG group 1), which is clearly visible in the surface plots (Fig 1). A reduction of antimicrobial costs has been reported as resulting in major savings in some studies [19,20]. In this study however, these savings were much lower because consumption was already relatively low and the antibiotics prescribed are mostly relatively inexpensive generic products. A 23% reduction for DRG Group 1 was observed, which showed a trend to significance, comprising 16% of the total costs (Table 1). Further studies with a financial evaluation were published, but only a few studies included more than savings from direct antimicrobial costs [13,21–24]. It is therefore difficult to compare economical studies for the effects of ASPs. Different parameters were taken into considerations, different methods and interventions were used and different types of patients were targeted for intervention. However, most intervention studies that included costs are reporting positive results [10,13,21,23,24].

The monetary costs and benefits in this study are for the most part indirect costs and savings because these are fixed costs in the hospital budget. Fixed costs are defined as costs that do not vary with the quantity of output in the short run, and include e.g. capital, equipment and staff [25]. Personnel costs will not change on the short run because of effects from this study as long as there is no mutation in staff. Thus direct benefits will be in the reallocation of resources. The same holds true for the personnel costs of the A-Team. No new staff was hired for the implementation of this study, but current staff members have been prioritizing there available time differently. Because this was a pilot project, the time spent on this project had been allocated from designated research time as a resource. There are thus opportunity costs, which is why personnel costs should not be underestimated. Furthermore, if the A-Team were to be implemented throughout the entire hospital, additional staff would be needed. Current staff would have insufficient time to do consultations for the whole hospital besides their normal duties. The mentioned savings in nursing time due to the IV-PO switch are indirect savings as well. For IV treatment we saw a drop of 73.47 minutes per patient in DRG Group 1, based upon an average time of around 64.83 minutes a nurse would need per day per patient. This average is comparable to earlier Dutch studies, as well as international studies [26–28]. Total nursing time is slightly higher in this study, because insertion, control, changing and removal of the IV catheter are also taken into account. Since there was no change in the total nursing staff, the budget did not change and current expenses remained the same. Reallocated resources is in this case were more available time for treatment of other patients. It is very likely that the quality of care, as well as infection prevention and control will benefit from that [29,30]. The reallocation of nursing time could in itself lead to further savings. This, however, falls outside the scope of this study. The results we see in the reduction of length of stay are indirect, as well. The major parts of the high price of a bed-day are fixed costs like equipment and housing. These costs do not change if patients stay less time in the hospital. Freeing up beds for additional admissions, however, does mean these resources will be available for additional patients and procedures. Thus, the benefits should create the possibility for increasing the revenue-generating activities and shortening waiting lists [31]. Effects might even be slightly higher when procedures were included. Since we estimated that during the last days of admission these costs would be limited, we chose to be more conservative and leave them out of the equation. This study also looked at the readmission rates between the intervention group and the historic cohort, but no significant differences were observed between the two groups (DRG group 1: 17% vs 12%; p = 0.244; DRG group 2: 11% vs 20%; p = 0.190). They were therefore not incorporated into the model.

Unfortunately not all costs were known and could be incorporated into the model. For example adverse reactions and complications are missing because they are not stored in an objective, usable manner. Also missing are the indirect costs from a societal perspective. Preferably a study should include all costs associated with the implementation of an ASP, direct and indirect [32]. However, indirect effects of the interventions and the costs associated with these effects are difficult to determine correctly with just one ASP intervention study on one ward. Inherent to the study and the model there are some uncertainties. DRG group 1 has some sub-optimal baseline parameters. However extensive additional analyses showed no influences of these parameters on the outcome measures. Uncertainties within the model were made clear by using the different sensitivity analyses for five variables as well as doing a probabilistic sensitivity analysis, which showed positive results within the whole 95% confidence interval.

One of the main expected results of correct antibiotic usage is the lowering resistance rate in the hospital. These lowered rates will most likely reduce the risk of acquiring nosocomial infections in the hospital. This in turn will most likely contribute to reducing the risk of an outbreak of such an infection, which will be accompanied by substantial costs [3]. As shown here, an A-Team on its own can already generate a positive revenue and ROI, by improving the usage of antibiotics. Assuming that this would subsequently lead to lowering the possible number of outbreaks, there are thus considerable additional indirect savings. However, the time-frame of this study was too short to examine effects on resistance rates. This is furthermore not only affected by an ASP, but also by for example hospital hygiene measures and other infection prevention measures, not only from the targeted hospital, but also from hospitals within the same health care network [33]. Such networks can exist regionally, but keeping in mind the European directive on international patient care and differences between e. g. MRSA bacteremia in Germany and the Netherlands, international borders should not be neglected [34]. Being an academic hospital, there are more patient movements to and from our hospital, making the hospital more prone to receive patients with (resistant) hospital acquired infections [35]. For a more extensive financial evaluation of ASPs and supplemental infection prevention measures, it would important that local and regional policies and measures are neglected. Including more data would make a model more precise, also from an economical point of view [36]. Further research should therefore be focused on the improvement of appropriate models. However, using our model, it is already possible to estimate at least some of the financial effects of an A-Team in any given hospital. Since this study is so far one of a few studies looking beyond direct antimicrobial costs, it hopefully will contribute to more in depth research into the financial effects of ASPs and A-Teams. For our hospital we can state that it is worth to invest in day-2 case-audits, because the reduction in LOS makes the program highly cost-effective.
